# Effects of Dextrose and Lipopolysaccharide on the Corrosion Behavior of a Ti-6Al-4V Alloy with a Smooth Surface or Treated with Double-Acid-Etching

**DOI:** 10.1371/journal.pone.0093377

**Published:** 2014-03-26

**Authors:** Leonardo P. Faverani, Wirley G. Assunção, Paulo Sérgio P. de Carvalho, Judy Chia-Chun Yuan, Cortino Sukotjo, Mathew T. Mathew, Valentim A. Barao

**Affiliations:** 1 Department of Dental Materials and Prosthodontics, Aracatuba Dental School, Universidade Estadual Paulista (UNESP), Aracatuba, São Paulo, Brazil; 2 Department of Surgery and Integrated Clinic, Aracatuba Dental School, Universidade Estadual Paulista (UNESP), Aracatuba, São Paulo, Brazil; 3 Department of Restorative Dentistry, University of Illinois at Chicago–College of Dentistry, Chicago, Illinois, United States of America; 4 Department of Orthopedic Surgery, Rush University Medical Center, Chicago, Illinois, United States of America; 5 Department of Prosthodontics and Periodontology, Piracicaba Dental School, Universidade of Campinas (UNICAMP), Piracicaba, São Paulo, Brazil; University of Akron, United States of America

## Abstract

Diabetes and infections are associated with a high risk of implant failure. However, the effects of such conditions on the electrochemical stability of titanium materials remain unclear. This study evaluated the corrosion behavior of a Ti-6Al-4V alloy, with a smooth surface or conditioned by double-acid-etching, in simulated body fluid with different concentrations of dextrose and lipopolysaccharide. For the electrochemical assay, the open-circuit-potential, electrochemical impedance spectroscopy, and potentiodynamic test were used. The disc surfaces were characterized by scanning electron microscopy and atomic force microscopy. Their surface roughness and Vickers microhardness were also tested. The quantitative data were analyzed by Pearson's correlation and independent *t*-tests (α = 0.05). In the corrosion parameters, there was a strong lipopolysaccharide correlation with the I_pass_ (passivation current density), C_dl_ (double-layer capacitance), and R_p_ (polarization resistance) values (*p*<0.05) for the Ti-6Al-4V alloy with surface treatment by double-acid-etching. The combination of dextrose and lipopolysaccharide was correlated with the I_corr_ (corrosion current density) and I_pass_ (*p*<0.05). The acid-treated groups showed a significant increase in C_dl_ values and reduced R_p_ values (*p*<0.05, *t*-test). According to the topography, there was an increase in surface roughness (R^2^ = 0.726, *p*<0.0001 for the smooth surface; R^2^ = 0.405, *p* = 0.036 for the double-acid-etching-treated surface). The microhardness of the smooth Ti-6Al-4V alloy decreased (*p*<0.05) and that of the treated Ti-6Al-4V alloy increased (*p*<0.0001). Atomic force microscopy showed changes in the microstructure of the Ti-6Al-4V alloy by increasing the surface thickness mainly in the group associated with dextrose and lipopolysaccharide. The combination of dextrose and lipopolysaccharide affected the corrosion behavior of the Ti-6Al-4V alloy surface treated with double-acid-etching. However, no dose-response corrosion behavior could be observed. These results suggest a greater susceptibility to corrosion of titanium implants in diabetic patients with associated infections.

## Introduction

Diabetes is a disorder marked by abnormal lipid and glucose metabolism, often with serious complications leading to premature death [1], and it is considered a public health concern worldwide [2]. According to the Centers for Disease Control and Prevention, 10.9 million (26.9%) people aged ≥65 years and 215,000 people younger than 20 years old had diabetes in the United States in 2010, with estimated annual treatment costs of $174 billion [3].

Periodontal diseases such as periodontitis are two and a half times more likely to occur in individuals with diabetes than in those without it [4]. Additionally, diabetes presents a stalemate to patients who undergo treatment with dental implants [5]. Hyperglycemia leads to overproduction of superoxide, which contributes to the pathogenesis of diabetic micro- and macrovascular complications [6]–[8], predisposing to at least a delay in peri-implant bone repair or causing peri-implantitis [7]–[10].

*Porphyromonas gingivalis* is the most common bacterium contributing to peri-implantitis in partially and completely edentulous patients [11]. *P. gingivalis* and other Gram-negative bacteria produce lipopolysaccharide (LPS), located in the bacterial cell walls [12], [13], and its presence dictates the prognosis of implant treatment [12]–[15].

Dental implants are composed mainly of titanium (Ti) and feature a strong bond with water or air molecules in the atmosphere, which promotes the immediate formation of a Ti oxide layer on the metal surface. This property creates proper surface energy for osseointegration and corrosion resistance [16], [17]. However, changes in the surrounding oral environment, such as pH and thermal oscillations, and the presence of biofilm, can degrade the oxide layer, allowing for the exchange of Ti ions. This activity contributes to the corrosion process of the implant surface [18]–[20], substantially affecting the mechanical properties and biocompatibility of the implants, resulting in the failure of rehabilitative treatment [21]–[24].

The corrosion resistance of medical and dental Ti implants has been evaluated *in vitro* in simulated physiological and systemic conditions [21], [23], [25]–[28]. However, few clinical studies have reported on the corrosion of Ti in dentistry. Olmedo et al. [29] reported 2 cases of injury reaction in the peri-implant mucosa, which suggested that it was caused by Ti particles released into the surrounding tissue, resulting in corrosion of the osseointegrated implant surface. In this context, 153 patients rehabilitated with single implants underwent biopsy of the mucosa adjacent to the implant cover screws during the period of osseointegration (4–6 months). In total, 41% of these patients showed Ti particles released from the implant surface, sometimes free or surrounded by macrophages, denoting foreign body reaction with probable metal corrosion [30].

Several mechanical and chemical modifications of dental implant surfaces – such as titanium plasma spray (TPS), hydroxyapatite coating, electrochemical and mechanical polishing, acid etching, aluminum oxide sandblasting, and laser irradiation – have been proposed. These surface treatments have increased the percentage of bone-implant contact, especially in low-bone-density areas, and have accelerated the osseointegration phenomenon [31]–[38].

Therefore, this study investigated the corrosion and microstructure behavior of Ti-6Al-4V alloys as a function of the Ti surface (smooth and modified by treatment with double-acid-etching) in simulated body fluid (SBF) with different dextrose (0 mM, 5 mM, 7.5 mM, and 15 mM) and LPS (0 μg/mL, 0.15 μg/mL, 15 μg/mL, and 150 μg/mL) concentrations, used alone or in combination.

The hypothesis was that the Ti-6Al-4V alloy surface treated with double-acid-etching would generate a corrosion pattern different from that observed in the smooth-surface alloy, both in the combination of dextrose and LPS as well as evaluated separately. Additionally, it was hypothesized that the presence of dextrose and LPS would decrease the corrosion resistance of the Ti-6Al-4V alloy.

## Materials and Methods

For the electrochemical test, 96 Ti-6Al-4V alloy discs (15 mm in diameter and 2 mm in thickness) were fabricated. The tested surfaces were detailed as below:

1 - Ti-6Al-4V alloy disc with smooth surface, mimicking implants with machined surface (n = 48); and

2 - Ti-6Al-4V alloy disc modified by treatment with double-acid-etching (n = 48).

### Disc preparation

The specimens were divided into 32 groups (n = 3) according to type of surface (smooth or etched with acid), dextrose concentration (0 mM, 5 mM, 7.5 mM, and 15 mM) (Sigma Chemical, St. Louis, MO, USA), and LPS concentration (0 μg/mL, 0.15 μg/mL, 15 μg/mL, and 150 μg/mL) (055: B5, Sigma Chemical) (Table 1). The discs were polished and cleaned by standard metallographic methods [23], [25]. Specimens were polished with sequential sandpaper of 320, 400, 600, and 800 grit (Carbimet 2, Buehler, Lake Bluff, IL, USA) in an automatic polisher (ECOMET 250PRO/AUTOMET 250, Buehler). Subsequently, a polishing cloth (TextMet Polishing Cloth, Buehler), diamond paste (MetaDi 9-micron, Buehler), and lubricant (MetaDi Fluid, Buehler) were used.

**Table 1:** Groups for testing corrosion in SBF as a function of the concentrations of dextrose (DEX), lipopolysaccharide (LPS), and the association of LPS and dextrose (DEXLPS) (n = 3).

Surface Types of Discs	Dextrose Concentration (mM)	Concentration of LPS (μg/mL)
**Smooth**		
SBF (control)	0	0
DEX5	5	0
DEX7.5	7.5	0
DEX15	15	0
LPS0.15	0	0.15
LPS15	0	15
LPS150	0	150
DEX5 LPS0.15	5	0.15
DEX5 LPS15	5	15
DEX5 LPS150	5	150
DEX7.5 LPS0.15	7.5	0.15
DEX7.5 LPS15	7.5	15
DEX7.5 LPS150	7.5	150
DEX15 LPS0.15	15	0.15
DEX15 LPS15	15	15
DEX15 LPS150	15	150
**Double-acid-etching**		
SBF (control)	0	0
DEX5	5	0
DEX7.5	7.5	0
DEX15	15	0
LPS0.15	0	0.15
LPS15	0	15
LPS150	0	150
DEX5 LPS0.15	5	0.15
DEX5 LPS15	5	15
DEX5 LPS150	5	150
DEX7.5 LPS0.15	7.5	0.15
DEX7.5 LPS15	7.5	15
DEX7.5 LPS150	7.5	150
DEX15 LPS0.15	15	0.15
DEX15 LPS15	15	15
DEX15 LPS150	15	150

Finally, specimens were mirror-finished with polishing cloth (Chemomet I, Buehler) and colloidal silica (MasterMeD, Buehler). The discs were ultrasonically cleaned with deionized water, de-greased with 70% propanol (Sigma Chemical) for 10 min, and dried with hot air at 250°C. The surface roughness of all samples was determined, to provide standardization in the finishes of the Ti-6Al-4V alloy discs [Roughness average (Ra) of 27.64±7.43 nm].

Dextrose at a concentration of 15 mM was selected as equivalent to 270 mg/dL of blood glucose, commonly found in patients with uncontrolled diabetes or in patients with undiagnosed type II diabetes [39]–[41]. Likewise, for the simulation of diabetes in the initial stages, the concentration of 7.5 mM dextrose was used, which corresponds to 135 mg/dL blood glucose. To simulate healthy patients with blood glucose levels within the normal range, the concentration of 5 mM dextrose was used, corresponding to 90 mg/dL blood glucose. Finally, as a control group, a zero concentration of dextrose was used for comparison among groups. The LPS concentrations (0 μg/mL, 0.15 μg/mL, 15 μg/mL, and 150 μg/mL) were used according to previous studies by Barao et al. [23] and Mathew et al. [42], to simulate the infection process.

### Discs with smooth surfaces

After the polishing protocol was applied to the Ti-6Al-4V alloy discs, 48 discs were randomly selected for the test corresponding to the smooth surface.

### Surface acid modifications

Forty-eight discs were subjected to surface modification according to market availability. To obtain the treated surface, we chemically treated the smooth discs with double-acid-etching (nitric, sulfuric, and hydrochloric acid), according to company proprietary standards (Military Institute of Engineering - IME, Rio de Janeiro, Brazil).

### Electrochemical test

The electrochemical test protocol followed that described previously in the literature [23], [25]. The tests were performed in an electrochemical cell made of polysulfone, which has 4 wells with an electrolyte capacity of 10 mL. All measurements were performed by a standardized method of three-cell electrodes according to the instructions of ASTM International [formerly known as the American Society for Testing and Materials (ASTM) (G61-86 and G31-72)]. A saturated calomel electrode (SCE) was used as the reference electrode (RE), a graphite rod as a counter-electrode (CE), and the exposed surface of the Ti-6Al-4V alloy disc as a working electrode (WE, exposed area of the smooth Ti  = 1.77 cm^2^, and Ti treated with double-acid-etching  = 2.62 cm^2^). A potentiostat (Interface 1000, Gamry Instruments, Warminster, PA, USA) connected to a computer was used to perform the corrosion measurements. A 10-mL quantity of electrolyte solution (SBF with or without dextrose and/or LPS) was used for each corrosion experiment [23], [25], [43]. The electrolyte for simulating blood plasma (SBF) has physiologically characteristic temperatures (37°C) and pH (7.4) [44] to mimic certain clinical conditions.

Initially, the Ti-6Al-4V alloy discs were subjected to a cathodic potential (−0.9 V vs. SCE) for 10 min to ensure the standardization of the oxide layer from the surface thereof. The open circuit potential (OCP) was monitored for a period of 3600 s to evaluate the potential of the material before application of the solution and to stabilize the system. Electrochemical impedance spectroscopy (EIS) was used to investigate the formation and growth of the oxide layer on the Ti surface and the properties of this film (corrosion kinetics). EIS, through the electrochemical process, can be represented by an equivalent electric circuit, and the oxide film properties (capacitance and resistance) were quantified to determine the corrosion process and related corrosion kinetics. The EIS measurements were performed at a frequency range of 100 KHz to 5 mHz, with an AC amplitude curve of 10 mV applied to the electrode at its corrosion potential (E_corr_) [23], [25], [42]. These values were used to determine the real (Z′) and imaginary (Z″) components of impedance, which were plotted in the Bode plot or total impedance (|Z|) and phase angle (theta). Finally, the specimens were anodically polarized from −1.2 V to 1.8 V at a scan rate of 2 mV/s.

The corrosion parameters were obtained by means of potentiodynamic polarization curves. Tafel's method was used to estimate the corrosion rate (corrosion current density - I_corr_) and the corrosion potential (E_corr_) of the Ti-6Al-4V alloy. The passivation current density (I_pass_) was the amount of current density in the transition between the active and passive regions in the polarization curve of the Ti-6Al-4V alloy. The EIS data were used to model the corrosion process (corrosion kinetics) and to describe the properties of the oxide film formed on the surfaces of the Ti-6Al-4V alloy discs. For this, we used Randle's circuit, in which the polarization resistance is in parallel with the capacitance of the double layer while in series with the resistance of solution (R_sol_). Based on such a modeling approach, an electrical circuit can be developed equivalent to the electrochemical reactions at the metal-solution interface. R_sol_ is the uncompensated electrolyte resistance between the reference and working electrodes; R_p_ is the polarization resistance or the charge transfer resistance at the interface between the working electrode and the electrolyte, in relation to the corrosion rate reactions at the passive field; and C_dl_ is the specific double-layer capacitance at the working electrode/electrolyte interface. The capacitance is represented by a constant-phase element (CPE) as an alternative to an ideal capacitance element, owing to the inhomogeneous passive layer at the material surface [25]. For the EIS simulation data (double-layer capacitance - C_dl_ and polarization resistance - R_p_), Gamry Echem Analyst software (Gamry Instruments) was used. A chi-square value (goodness of fit) less than 0.001 was considered to indicate an excellent agreement between the experimental data and the fitted values [45].

### Surface characterization

The evaluation of the corroded surfaces plays an important role in our understanding of the mechanisms of degradation. Several techniques for surface characterization were used as described below.

### Atomic Force Microscopy

The Ti-6Al-4V alloy discs with a smooth surface and surface-treated by acid-etching were analyzed by atomic force microscopy (AFM) (AFM, Veeco Metrology Inc., Santa Barbara, CA, USA). The images were transported from the microscope to a computer, and in the NanoScope Analysis program (2004 Veeco Instruments Inc., Santa Barbara, CA, USA), the images were initially subjected to filters (lowpass and medium). Images in 3 dimensions (3D) were then obtained, and to facilitate a visual comparative analysis among the groups, a standardized scale of the z-axis was used.

### Scanning Electron Microscopy and Energy-dispersive Spectroscopy

Scanning electron microscopy (SEM) (JEOL, model JSM-7401F, Portland, OR, USA) was used to characterize possible irregularities on the surfaces of Ti-6Al-4V alloy discs. Comparisons were made between the images obtained from the Ti surfaces by the pre- and post-corrosion analyses of the tested electrolytes.

In addition, elemental chemical analyses were performed in small volumes, in the order of 1 μm^3^, through the technique of energy-dispersive spectroscopy (EDS) with a spectrometer. This allows for the simultaneous observation of the entire x-ray spectrum, which permits the rapid quantitative analysis (mapping) of the main elements of the Ti surfaces. Comparisons were made of the chemical composition of the different surfaces analyzed prior to and after the corrosion test.

### Analysis of surface roughness

The surface roughness (Ra - arithmetical mean surface roughness) of Ti-6Al-4V alloy was investigated before (baseline) and after the corrosion process by means of a profilometer (Dektak 150-d; Veeco, Plainview, NY, USA). Each specimen was individually placed in the center of the equipment, with the measuring tip of the profilometer on the specimen surface. Three measurements were performed at different areas on each disc (0.25 mm cut-off at a speed of 0.05 mm/s), and the mean value was calculated [46].

### Microhardness analysis

The microhardness of the Ti-6Al-4V alloy discs was verified at baseline (before corrosion tests) and after the corrosion tests by means of a microhardness tester (Shimadzu HMV-2000 Micro Hardness Tester, Shimadzu Corporation, Kyoto, Japan). This measurement was performed at room temperature (22±2°C). The applied load was 500 gf for 15 s, and the microhardness was expressed in units of Vickers Hardness (VHN) [47], [48]. The values of Vickers hardness were calculated according to the following formula: VHN 2P  =  sin (136°/2)/d2, where P =  applied load and d =  length of the diagonals of indentations. The test was repeated 4 times in 4 randomly distributed points on the surface of each disc. The average of these 4 replicates corresponded to the value of the Vickers microhardness.

### Statistical Analysis

Pearson's correlations were used to identify the relationships between the corrosion parameters (E_corr_, I_corr_, I_pass_, C_dl_, and R_p_) and the concentrations of LPS or dextrose (used alone or combined). Correlations of roughness and microhardness vs concentrations of LPS or dextrose (used alone or combined) were also investigated.

The independent *t*-test was used to compare the two Ti surface conditions (smooth or treated with double-acid-etching) for all parameters described above. All tests were conducted with a significance level of 5% (SPSS version 17.0 - Statistical Package for the Social Sciences, Inc., Chicago, IL, USA).

In this study, 3 specimens were used per group, with a power effect of 0.873 (Cohen effect size statistics).

## Results

### Electrochemical data

**Figure 1:**
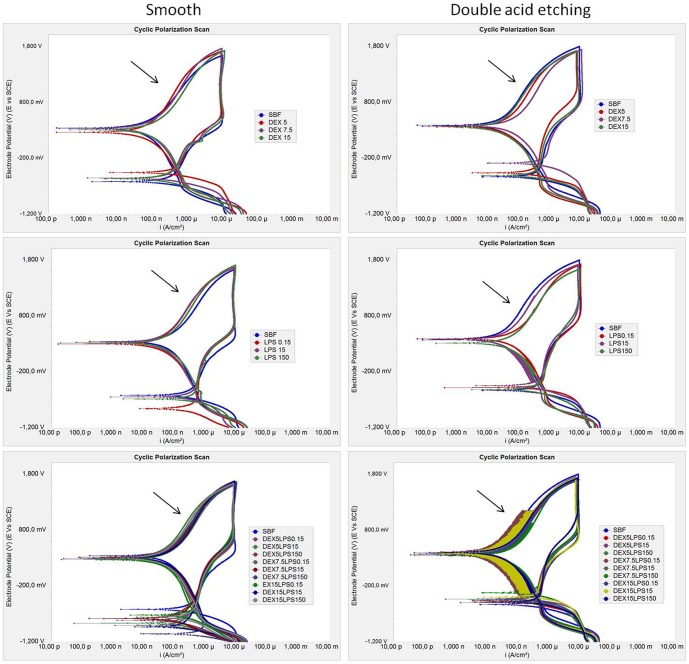
Representative cyclic potentiodynamic polarization curve of Ti-6Al-4V alloy. Tests were conducted in simulated body fluid - SBF (control) with different concentrations of dextrose (DEX), lipopolysaccharide (LPS), and combinations of dextrose and lipopolysaccharide (DEXLPS).

**Figure 2:**
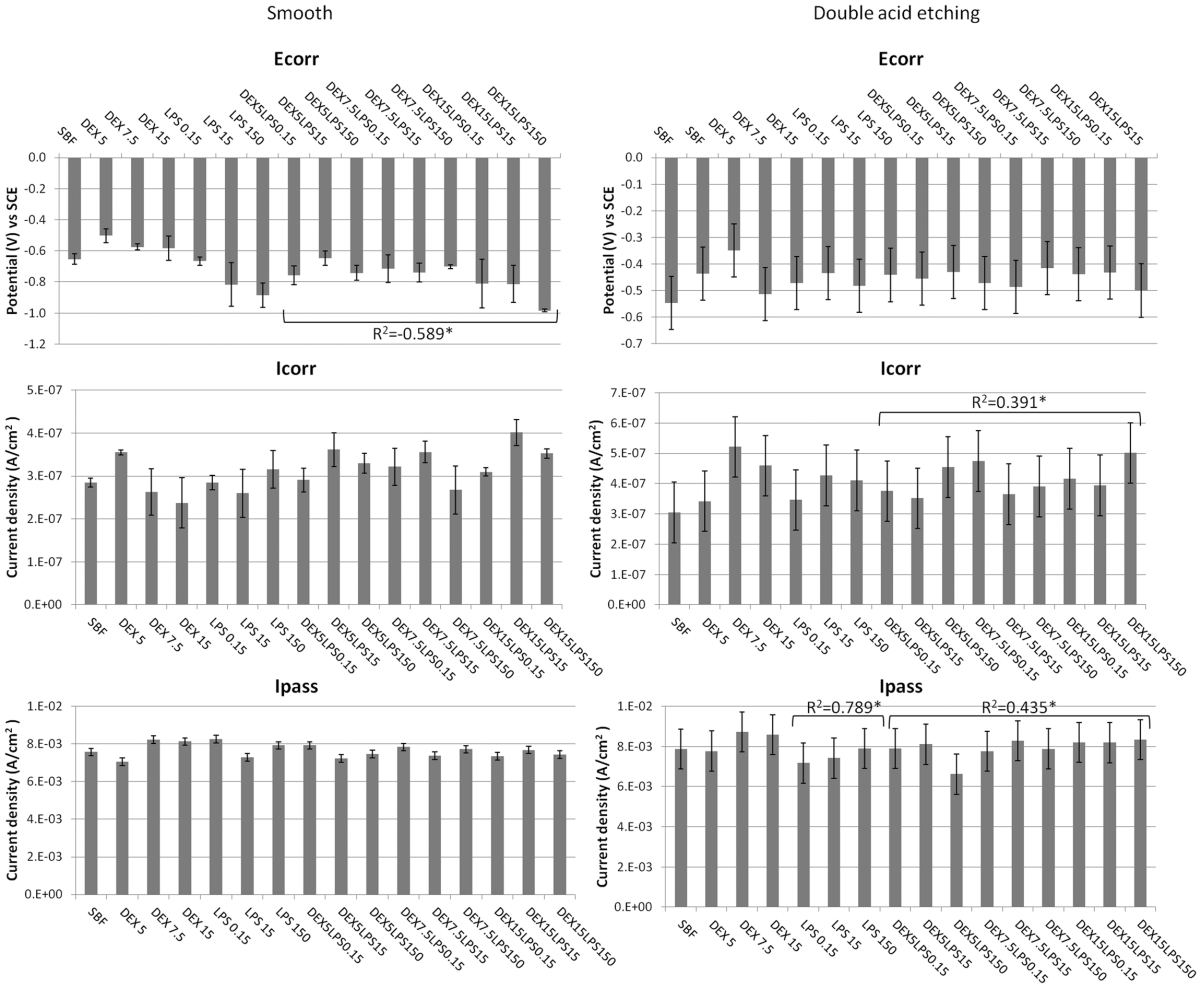
Electrochemical data of cyclic potentiodynamic polarization curve. Mean and standard deviation of corrosion potential (E_corr_), corrosion current density (I_corr_), and the passivation current density (I_pass_) for the Ti-6Al-4V alloys with a smooth surface and etched with acid, in SBF with different concentrations of dextrose (DEX) and lipopolysaccharide (LPS), alone or in combination (DEXLPS). * Denotes significant correlation at the 0.05 level.

**Figure 3:**
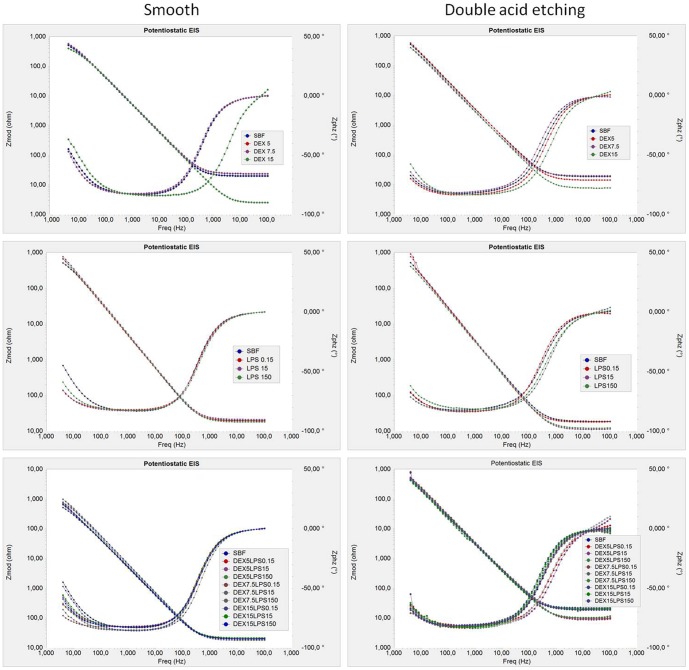
Electrochemical impedance spectroscopy (EIS) represented by the Bode plot of the Ti-6Al-4V alloy. Tests were conducted in simulated body fluid - SBF (control) with different concentrations of dextrose (DEX), lipopolysaccharide (LPS), and combinations of dextrose and lipopolysaccharide (DEXLPS).

**Figure 4:**
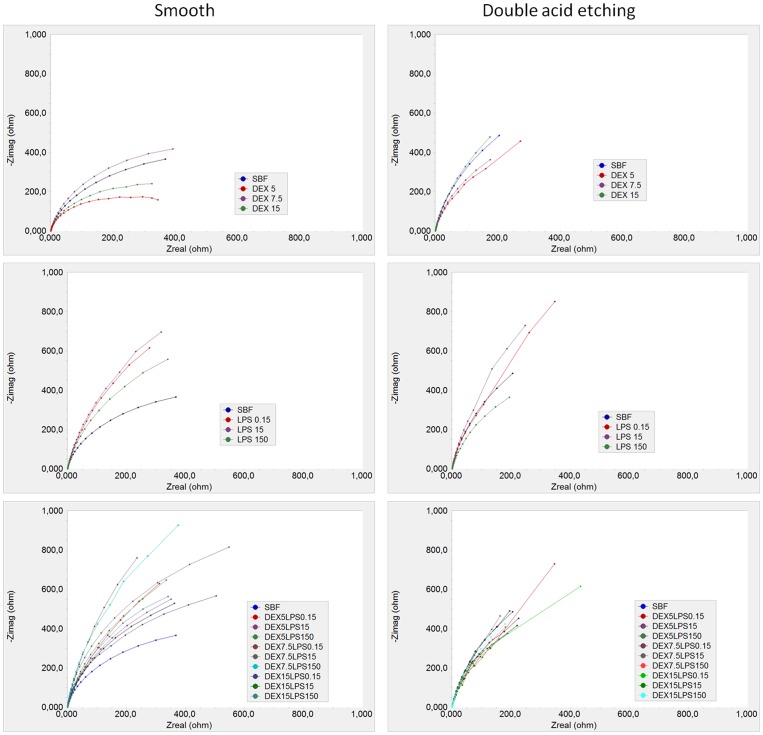
Electrochemical impedance spectroscopy (EIS) represented by the Nyquist plot of the Ti-6Al-4V alloy. Tests were conducted in simulated body fluid - SBF (control) with different concentrations of dextrose (DEX), lipopolysaccharide (LPS), and combinations of dextrose and lipopolysaccharide (DEXLPS).

**Figure 5:**
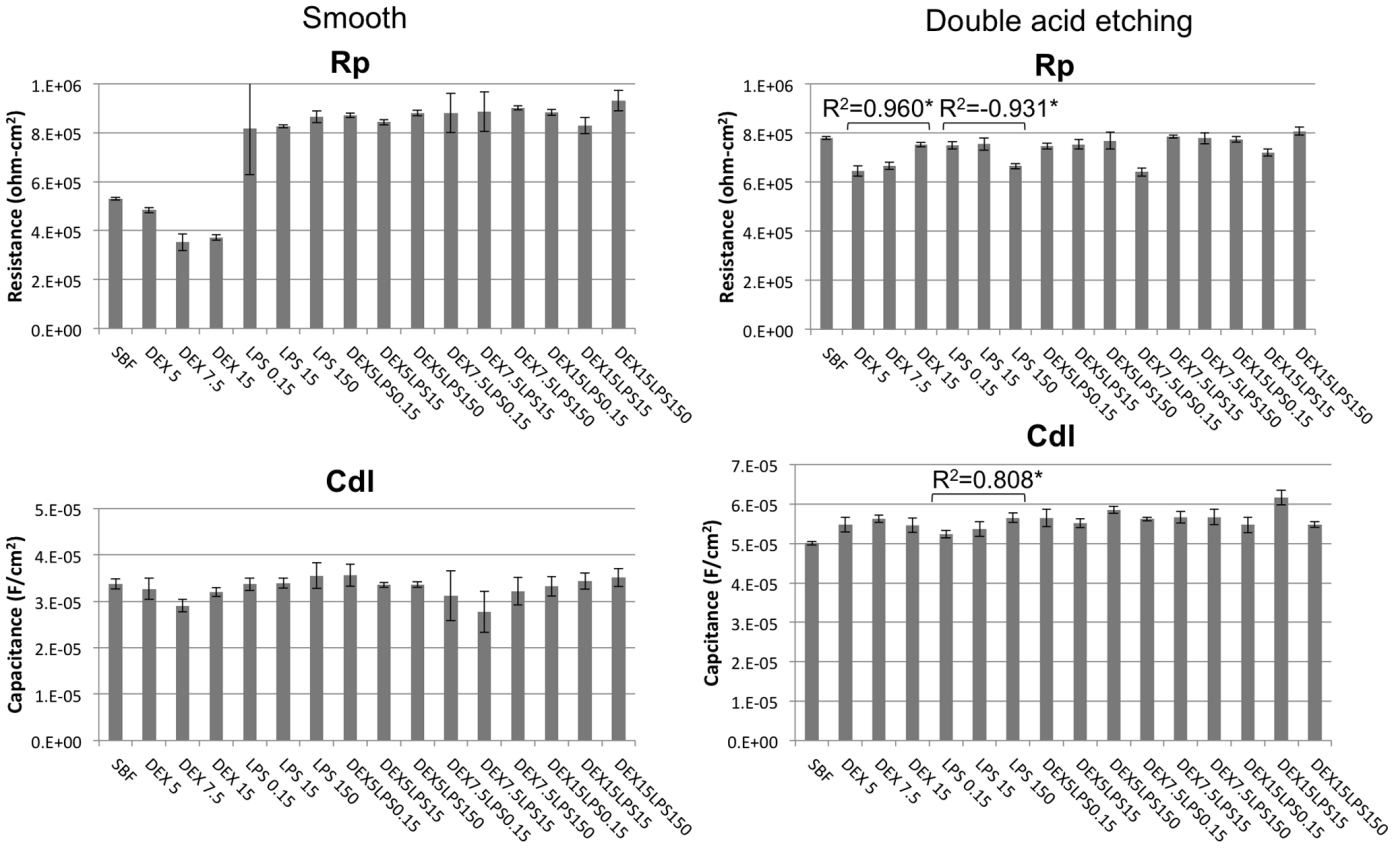
Electrochemical data from the EIS test. Means and standard deviations of polarization resistance (R_p_) and capacitance (C_dl_) for the Ti-6Al-4V alloys with a smooth surface and etched with acid in SBF with different concentrations of dextrose (DEX) and lipopolysaccharide (LPS), alone or in combination (DEXLPS). * Denotes significant correlation at the 0.05 level.

The cyclic potentiodynamic polarization curves, the electrochemical data, and EIS can be seen in Figs. 1 to 5. Regions of active-passive transition were observed in the cyclic polarization curves of the Ti-6Al-4V alloys in the control group (SBF) and for all tested groups (Fig 1). A typical passive plateau, which is common in the case of Ti, was evident with both surfaces (smooth and acid). The formation of negative hysteresis was obvious in the curves, as indicated by arrows, which enables us to state that the passive layer formed is strong and uniform, and that Ti in the presence of dextrose and/or LPS is not susceptible to corrosion by pits or cracks.

For the Ti-6Al-4V alloy with the smooth surface, only the association with dextrose and LPS (DEXLPS) was significantly correlated with some parameter of corrosion. A negative correlation with E_corr_ was noted (R^2^  = -0.589, *p*<0.001) (Table 2). For the Ti-6Al-4V alloy surface conditioned with double-acid-etching, the presence of dextrose was significantly correlated with R_p_ (R^2^ = 0.960, *p*<0.01) (Table 2). The LPS was correlated with the I_pass_ (R^2^ = 0.789, *p* = 0.011), C_dl_ (R^2^ = 0.808, *p* = 0.008), and R_p_ (R^2^ = -0.931, *p*<0.01) (Table 2). The combination of dextrose and LPS was correlated with I_corr_ (R^2^ = 0.391, *p* = 0.044) and I_pass_ (R^2^ = 0.435, *p* = 0.023) (Table 2).

**Table 2:** Correlations between parameters of corrosion, roughness, and hardness with concentrations of dextrose (DEX) and lipopolysaccharide (LPS) (used alone or together) for Ti-6Al-4V alloys with smooth surfaces and conditioned with double-acid-etching.

	Smooth						Double-acid-etching					
	Dextrose		LPS		Dextrose+LPS		Dextrose		LPS		Dextrose+LPS	
variable	R^2^	*P*	R^2^	*P*	R^2^	*P*	R^2^	*P*	R^2^	*P*	R^2^	*P*
E_corr_	−0.479	0.192	−0.615	0.078	−0.589*	0.001	0.639	0.064	−0.246	0.523	−0.121	0.547
I_corr_	−0.664	0.051	0.469	0.203	0.250	0.208	0.382	0.311	0.347	0.361	0.391*	0.044
I_pass_	0.534	0.139	0.069	0.860	−0.134	0.504	0.500	0.170	0.789*	0.011	0.435*	0.023
C_dl_	0.070	0.858	0.477	0.194	0.008	0.967	−0.172	0.658	0.808*	0.008	0.104	0.605
R_p_	−0.557	0.119	0.224	0.562	0.217	0.277	0.960*	0.0001	−0.931*	0.0001	0.259	0.193
Roughness	0.0001	1.000	0.105	0.788	0.726*	0.0001	−0.326	0.392	0.601	0.087	0.405*	0.036
Microhardness	−0.551	0.124	0.218	0.573	−0.915*	0.0001	−0.342	0.368	0.175	0.653	0.781*	0.0001

* Correlation is significant at the 0.05 level (2-tailed).

The double-acid-etching surface treatment significantly increased the I_corr_ values for most groups (*p*<0.05, *t*-test) (Table 3, Fig 2). The passivation of the groups treated with double-acid-etching tended to be delayed, a fact evidenced by the values of I_pass_. Conversely, the E_corr_ values were lower for the Ti-6Al-4V alloys with the smooth surface (Table 3, Fig 2).

**Table 3:** Comparisons (independent *t*-test) between the Ti-6Al-4V alloys with smooth surfaces and double-acid-etching parameters for corrosion, roughness, and microhardness.

	Variables						
Groups	E_corr_	I_corr_	I_pass_	C_dl_	R_p_	Roughness	Microhardness
**Baseline**	-	-	-	-	-	0.015*	0.127
**SBF (control)**	0.008*	0.123	0.040*	0.0001*	0.0001*	0.0001*	0.168
**DEX5**	0.083	0.161	0.105	0.0001*	0.0001*	0.002*	0.070
**DEX7.5**	0.002*	0.004	0.162	0.0001*	0.0001*	0.001*	0.0001*
**DEX15**	0.221	0.003*	0.186	0.0001*	0.0001*	0.0001*	0.005*
**LPS0.15**	0.019*	0.022*	0.024*	0.0001*	0.565	0.0001*	0.042*
**LPS15**	0.010*	0.012*	0.677	0.0001*	0.009*	0.0001*	0.259
**LPS150**	0.001*	0.027*	0.897	0.0001*	0.0001*	0.0001*	0.093
**DEX5 LPS0.15**	0.001*	0.008*	0.908	0.0001*	0.0001*	0.008*	0.747
**DEX5 LPS15**	0.003*	0.776	0.010*	0.0001*	0.003*	0.001*	0.391
**DEX5 LPS150**	0.002*	0.002*	0.001*	0.0001*	0.006*	0.0001*	0.161
**DEX7.5 LPS0.15**	0.014*	0.004*	0.652	0.001*	0.007*	0.0001*	0.023*
**DEX7.5 LPS15**	0.003*	0.726	0.029*	0.0001*	0.098	0.001*	0.0001*
**DEX7.5 LPS150**	0.003*	0.019*	0.215	0.0001*	0.001*	0.0001*	0.001*
**DEX15 LPS0.15**	0.015*	0.001*	0.009*	0.0001*	0.0001*	0.0001*	0.0001*
**DEX15 LPS15**	0.006*	0.725	0.010	0.0001*	0.006*	0.0001*	0.0001*
**DEX15 LPS150**	0.0001*	0.0001*	0.007*	0.0001*	0.009*	0.0001*	0.0001*

* Significant at the 0.05 level (independent *t*-–test).

The kinetics of corrosion and passive film formation was demonstrated through the Bode plot (impedance IZI vs frequency; and phase angle vs frequency) (Fig 3). In the phase angle, only a time constant was observed for all groups, which indicates the formation of a compact Ti oxide film, homogeneous and a shield to the metal surface. In high and low frequencies, the overall impedance values were lower in the presence of 15 mM dextrose, for both the smooth-surface acid-treated Ti-6Al-4V alloys. In general, the Nyquist plot (Zimg vs Zreal) (Fig 4) showed that the dextrose and LPS alone or combined decreased the semicircular diameter of the capacitance loop for both Ti-6Al-4V alloy surfaces compared with the control group (SBF). This suggests a lower corrosion resistance of the Ti in these situations. LPS increased the semicircular diameter of the capacitance loop of the smooth Ti-6Al-4V alloy (Fig 4).

The type of Ti-6Al-4V alloy surface exerted a strong influence on the corrosion kinetics, as observed in the values of C_dl_ and R_p_. The groups treated with double-acid-etching showed a significant increase in C_dl_ values and reduced values of R_p_ (*p*<0.05, *t*-test), except for the control group and the dextrose groups, in which the smooth surface produced greater R_p_ values (Table 3, Fig 5).

### Topographic data

**Figure 6:**
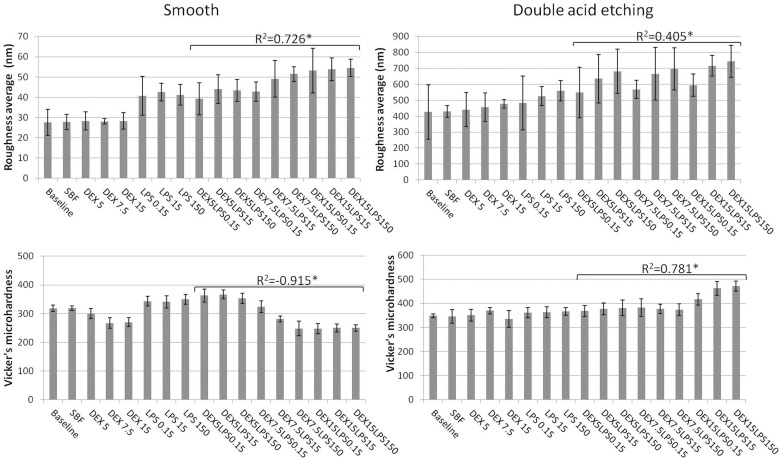
Data on surface topography. Means and standard deviations of surface roughness (Ra) (in nm) and Vickers microhardness for the Ti-6Al-4V alloys with a smooth surface and those etched with acid in SBF with different concentrations of dextrose (DEX) and lipopolysaccharide (LPS), alone or in combination (DEXLPS). * Denotes significant correlation at the 0.05 level.

The average surface roughness (Ra) and microhardness values are shown in Fig 6. Only the association of dextrose and LPS showed a correlation with the topographic data for both types of Ti-6Al-4V alloy surfaces. A positive correlation with the Ra parameter was noted (R^2^ = 0.726, *p*<0.0001 for the smooth Ti; R^2^ = 0.405, *p* = 0.036 for the acid Ti). There was a negative correlation for the microhardness data (R^2^ = −0.915, *p*<0.0001) in the smooth Ti, while for the Ti with double-acid-etching, a positive correlation was noted (R^2^ = 0.781, *p*<0.0001).

When the two surface conditions of the Ti-6Al-4V alloy are compared, it can be noted that the double-acid-etching treatment resulted in a significant increase (*p*<0.05, t-test) in the surface roughness of the material for all groups (Table 3, Fig 6). Overall, the Ti-6Al-4V alloy with a smooth surface showed lower microhardness values when compared with the Ti-6Al-4V surface treated by double-acid-etching (Table 3, Fig 6).

**Figure 7:**
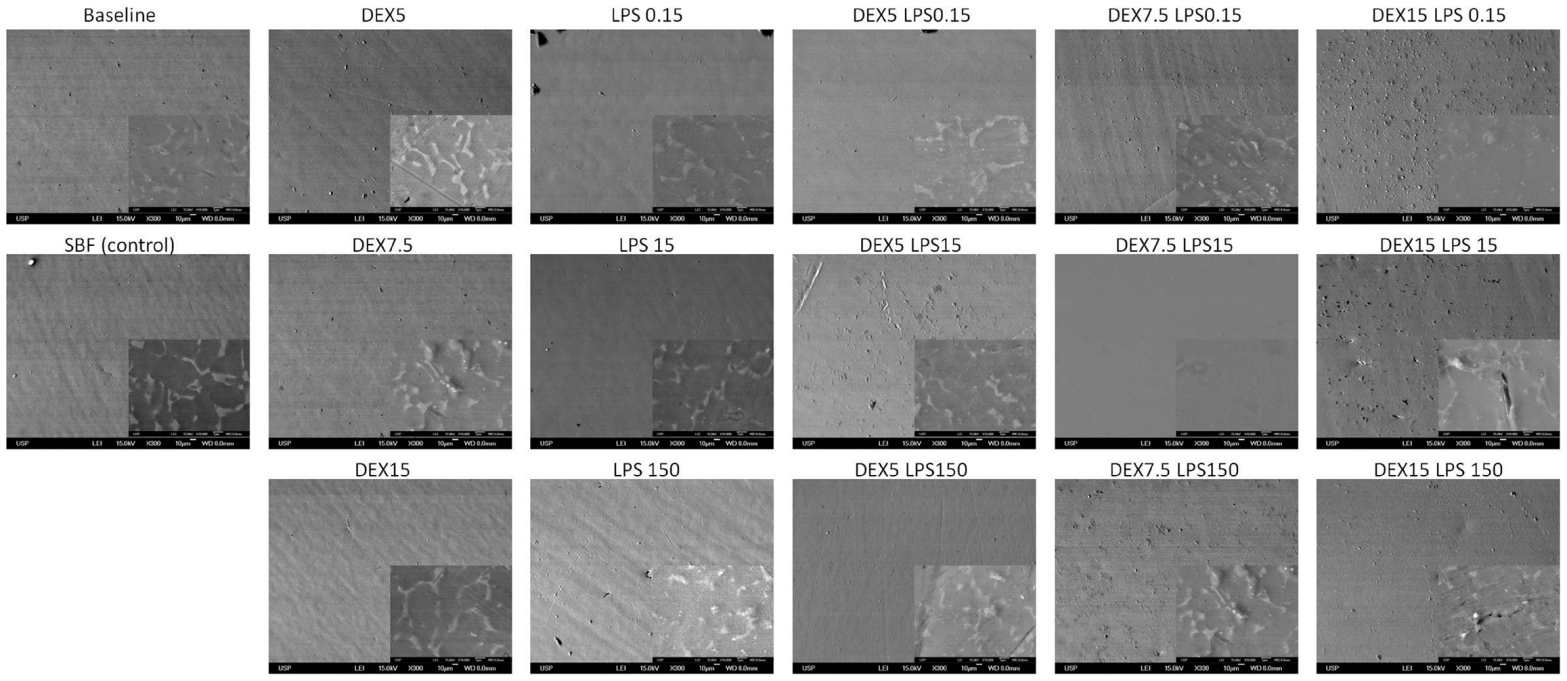
Scanning electron microscope images (300× and 10,000×) of the Ti-6Al-4V alloy with smooth surface, showing the Ti-6Al-4V alloy surface before and after corrosion in SBF (control - Co) with different concentrations of dextrose (DEX) and lipopolysaccharide (LPS), alone or in combination (DEXLPS). WD  =  8 mm; 15 Kv.

**Figure 8:**
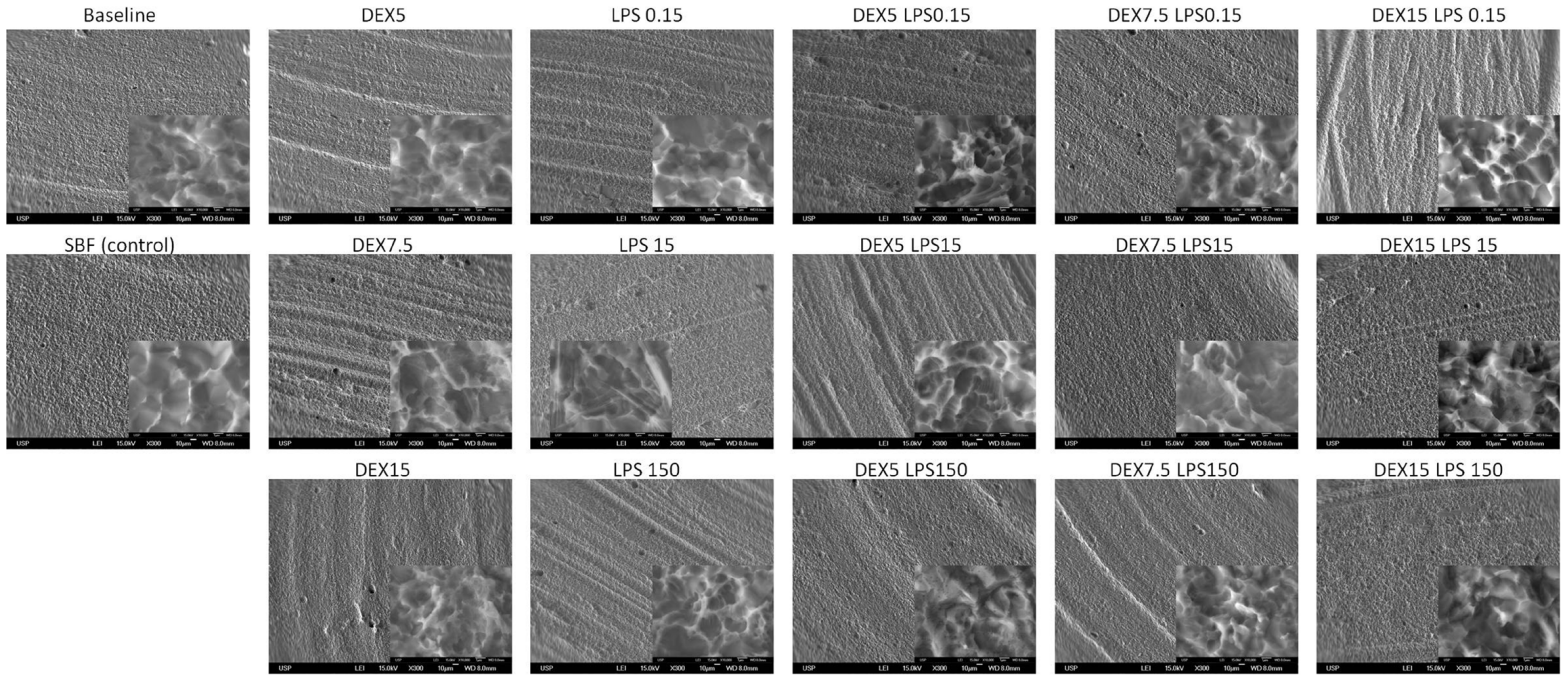
Scanning electron microscope images (300× and 10,000×) of the Ti-6Al-4V alloy with acid-etched surface, showing the Ti-6Al-4V alloy surface before and after corrosion in SBF (control - Co) with different concentrations of dextrose (DEX) and lipopolysaccharide (LPS), alone or in combination (DEXLPS). WD  =  8 mm; 15 Kv.

**Figure 9:**
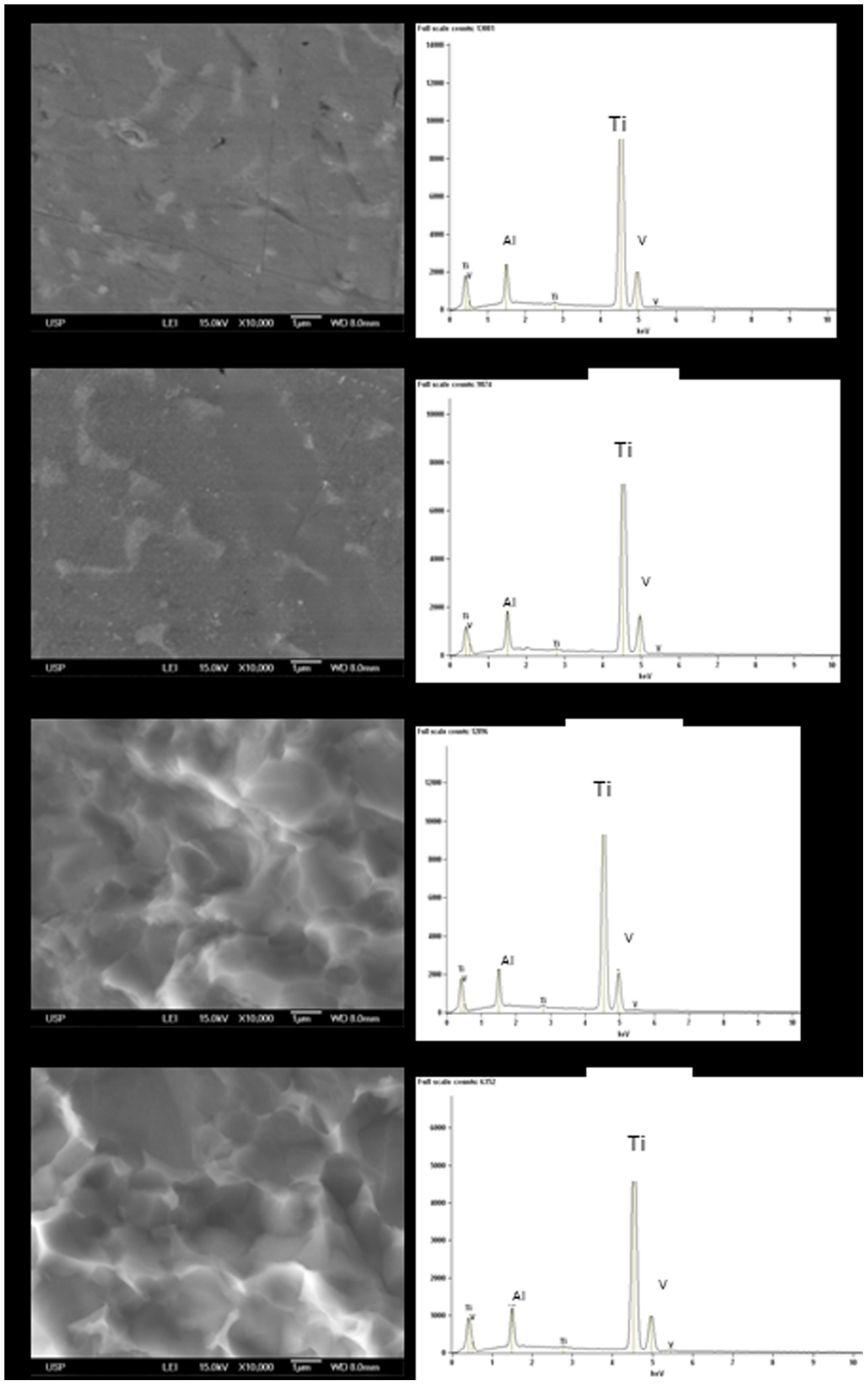
Representative energy-dispersive spectroscopy (EDS). (a) Ti-6Al-4V alloy with smooth surface before corrosion test. (b) Ti-6Al-4V alloy with smooth surface after the corrosion test. (c) Ti-6Al-4V with acid-etched surface before the corrosion test. (d) Ti-6Al-4V with acid-etched surface after the corrosion test.

The SEM images of the Ti-6Al-4V alloy with a smooth surface showed no changes in the microstructure of the Ti, except in the dextrose group (at a concentration of 15 mM) associated with LPS (concentrations of 15 and 150 μg/mL), which presented inlays, probably due to the substrates used (dextrose and/or LPS) (Fig 7). For the Ti-6Al-4V alloy treated with double-acid-etching, the comparison between the alloys at baseline and after the corrosion test did not show any change among the groups tested (Fig 8). The smooth Ti-6Al-4V alloy possessed a smooth and fairly flat surface (magnifications of 300× and 10,000×). The white spots identified mainly at the 10,000× magnification are due to the presence of aluminum in the Ti-6Al-4V alloy, confirmed by EDS (Fig 9). The Ti surface treated with acid showed striations on the entire surface, with rough microstructure quite evident. EDS analysis of the elementary chemical composition of the Ti-6Al-4V alloy, in two stages of the experiment (baseline and after the corrosion test), showed no change in the constituents of the Ti-6Al-4V alloy (Fig 9).

**Figure 10:**
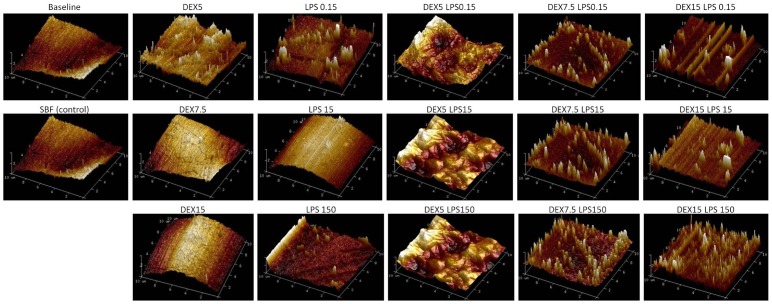
3D AFM images of a Ti-6Al-4V alloy with a smooth surface. Surfaces were characterized before (baseline) and after corrosion in SBF (control) with different concentrations of dextrose (DEX) and lipopolysaccharide (LPS), alone or in combination (DEXLPS).

**Figure 11:**
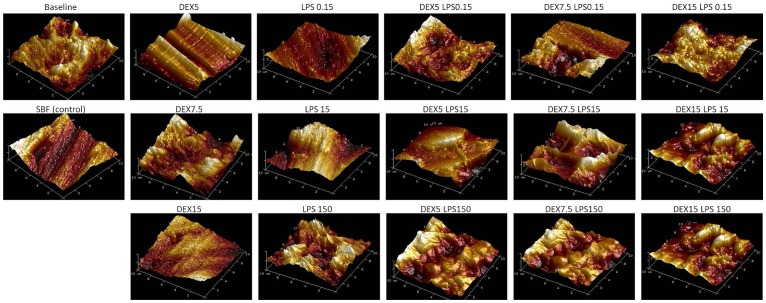
3D AFM images of the Ti-6Al-4V alloy with an acid-etched surface. Surfaces were characterized before (baseline) and after corrosion in SBF (control) with different concentrations of dextrose (DEX) and lipopolysaccharide (LPS), alone or in association (DEXLPS).

AFM analysis of the three-dimensional surface of the Ti-6Al-4V alloy demonstrated superficial changes in the material after the corrosion test for both the smooth-surface discs and those conditioned by acid. A greater apparent oxide thickness in the groups associated with dextrose and LPS (DEXLPS) was noted (Figs 10, 11).

## Discussion

The research hypotheses – that (1) the Ti-6Al-4V alloy with the surface treated with double-acid-etching generates a corrosion pattern different from that observed in the smooth surface, and that (2) the presence of LPS and dextrose decreases the corrosion resistance of the Ti-6Al-4V alloy – were partially accepted. The combination of dextrose and LPS negatively affected the electrochemical stability of the Ti-6Al-4V alloy treated with double-acid-etching. Dextrose alone did not impair the corrosion behavior of Ti-6Al-4V alloy for both surface conditions. The Ti-6Al-4V alloy with surface modification by acid etching showed a higher tendency toward corrosion.

### Corrosion behavior

In spite of the results obtained by the parameters of corrosion, a significant decrease (more electronegative) in the corrosion potential E_corr_ (*p*<0.01) was noticed on the smooth Ti-6Al-4V alloy when exposed to dextrose and LPS (DEX-LPS). The higher the concentration of the combined DEX-LPS, the lower the corrosion potential of the smooth Ti. The reduced values (more electronegative) of E_corr_ suggest that the smooth Ti provides a less stable surface [21], [22] that may be a result of decreased surface passivity. A previous study related the passivity to the thickening of the oxide/hydroxide layer in the titanium/electrolyte interface [21]. The isolated presence of dextrose and LPS alone had no correlation with the E_corr_ values of the smooth Ti-6Al-4V alloy at any of the concentrations tested (*p*>0.05).

Messer et al. [22] evaluated the electrochemical behavior of commercially pure Ti implants (cpTi) in inflammatory and hyperglycemic conditions. The corrosion potential shifted to noble values at high concentrations of dextrose (15 mM) at the same concentration used in this study, as a function of monocyte cultures and blood cells. However, no change in the corrosion rate was found when phosphate-buffered saline (PBS) was used as the medium. Hence it is believed that such inconsistency occurred due to the differences in the electrolyte used. Messer et al. used cultured blood cells and PBS. In the present study, SBF was used, and this medium has a composition similar to that of blood plasma, which may simulate a real clinical situation. Additionally, our tests were conducted over a short term, and Ti-6Al-4V was used instead of cpTi.

The addition of dextrose to the SBF tended to reduce the R_p_ values for smooth and acid Ti samples. However, the concentration of dextrose was positively correlated to the R_p_ in the Ti-6Al-4V alloy treated with double-acid-etching (*p*<0.01). This indicates that the resistance of the material increased with increasing concentrations of dextrose, showing similar R_p_ values at 15 mM of dextrose concentration when compared with SBF (control), thus protecting the surface of the material. It is believed that the saccharides and proteins on dental implant surfaces can enhance the corrosion resistance of the Ti in accordance with the binding affinity to the specific proteins and cause the release of metal ions from the surface of the material to the peri-implant region [21], [22]. However, the interaction of the metals and proteins, blood cells, and their products to promote corrosion depends on several factors, such as roughness, surface charge, material composition, and the affinity agent molecules of the metal [49], and may even protect the surface of the Ti by protein absorption through the electrolyte [21].

It was noted that the values of capacitance increased and the resistance values decreased with the increased concentration of LPS for Ti-6Al-4V alloys treated with acid (negative action of the LPS on the corrosion kinetics of the acid Ti). Furthermore, there was delayed passivation with the increasing concentration of LPS, due to a positive correlation for the I_pass_. Dextrose alone did not cause any effect on the corrosive properties of the Ti-6Al-4V alloy. In contrast, when dextrose was added to LPS (DEXLPS), there was an increase in the rate of corrosion current and less potential for passivation of Ti, confirming the deleterious effect of the LPS on the surface of Ti with acid treatment.

In this context, the action of LPS on the corrosion [23] and tribocorrosion [42] behavior of the Ti was evaluated *in vitro*. These authors stated that the polysaccharide portion of the LPS can induce the release of Ti ions, proven by the corrosion kinetics, through the values of C_dl_ and R_p_. This probably is due to a greater surface area for electrochemical reactions in the Ti-6Al-4V alloy treated with double-acid-etching. The results of this present study contradict those obtained by Messer et al. [21], who evaluated the Ti surface modified with oxide and phosphate and observed lower corrosion rates due to inflammatory and hyperglycemic conditions (concentration  =  1 μg/mL of LPS). It is believed that the concentration of LPS used in the Messer study [21], in simulating the inflammatory effects, was not able to affect the corrosive properties of the Ti. Herein, the concentrations of LPS (15 and 150 μg/mL) mimicked infections, and undoubtedly could cause more harmful effects on the Ti surface. In addition, because the smooth surface had a smaller contact area with the LPS, there was less connection with the polysaccharide portion of the Ti surface. This may have been responsible for the increased R_p_ values observed on the smooth Ti surface.

The use of LPS at concentrations that mimic infection with the smooth cpTi surface has been verified previously [23]. It was observed that increasing the concentration of LPS caused a significant increase in the rate of corrosion of the cpTi in artificial saliva. This inconsistency with the present study is mainly because simulated body fluid was used as an electrolyte, and the material tested was the Ti-6Al-4V alloy. Therefore, it is clear that the corrosion behavior of the Ti is dependent on the electrolyte and, to a certain extent, on its composition.

### Surface Topography

Reports in the literature agree that the corrosion process can increase the surface roughness of Ti [20], [42], [50]. Changes in Ti roughness may imply greater adherence of microorganisms [51]. Additionally, Barao et al. [52] inferred that the corrosion process increased the attachment of *P. gingivalis* on Ti substrata, increasing the tendency for peri-implantitis [53] and failure of the Ti implant. These findings are in agreement with the results from the current study with respect to the roughness values. In the smooth and treated Ti surfaces, there was a strong positive correlation for the combination of dextrose and LPS (DEXLPS), corroborating the parameters of corrosion.

The increase in Ra values with dextrose and LPS were visually confirmed by the 3D AFM images. This was especially observed in the smooth Ti-6Al-4V alloy by increases in the apparent oxide thickness. SEM images did not show great changes on the Ti-6Al-4V surfaces. Faverani et al. [46] showed that only substantial deterioration can be evidenced on the Ti-6Al-4V alloy surface. Yet, changes in the microstructure of the Ti-6Al-4V alloy in combination with dextrose and LPS were probably due to the impregnation of the substrates used herein.

Analysis of the microhardness data showed a strong negative correlation for the smooth Ti-6Al-4V alloy and a positive correlation for the Ti-6Al-4V alloy treated with acid, in the presence of dextrose combined with LPS. Yang et al. [54] also observed an increase in the microhardness of cpTi with surface treatment when compared with the smooth Ti. The lattice deformation and the solution hardening during surface treatment may be the driving force in such data [55]. Clinically, the surface hardening may be endorsed for load-bearing implants and dental implants [54]. There is still no scientific support to explain why the combination of dextrose and LPS during corrosion increased the surface hardness of the surface-treated group. Therefore, further studies should focus on this topic. It is important to highlight that the greater addition of sugars (i.e., dextrose) and LPS certainly raises the failure rates of dental implants [20], [51], [53] in the clinical setting.

### Clinical Implications

The *in vitro* simulations of this study showed that patients with a combination of hyperglycemia and infections might be more prone to experience implant failure, due to the reduced electrochemical stability of the Ti-6Al-4V alloy. It is noteworthy that it is not only diabetes entails hyperglycemia. Other systemic disorders also cause elevation of blood glucose levels, such as pancreatitis [56], [57], pancreatic cancer [57]–[59], Addison's disease, Cushing's syndrome, and other alterations of the adrenal glands [60]–[62]. Thus, it can be inferred that the blood glucose levels of patients undergoing dental implant procedures should be within normal limits. Furthermore, in view of the increased surface roughness and higher R_p_ values for the ‘isolated dextrose’ group, the hyperglycemic individuals must have good oral hygiene to reduce local biofilm accumulation.

### Limitations and future studies

In this study, the static effects of dextrose, LPS, and the combination of dextrose and LPS in simulated body fluid were investigated. However, it is known that the peri-implant environment is constantly subjected to the action of mechanical oscillations, especially during masticatory load [63], [64]. Thus, as suggested by Mathew et al. [42], future studies evaluating the linking of corrosion and wear (tribocorrosion) are warranted.

Moreover, the occurrence of peri-implantitis, bacteremia, or hyperglycemia in individuals with diabetes may be long-lasting; however, our electrochemical tests were carried out over the short term. Further studies to evaluate the effects of these conditions in the long term (for example, 24 and 48 h after immersion) should be performed.

## Conclusions

The combination of dextrose and LPS has a slight influence on the corrosion current density of the Ti-6Al-4V alloy treated with double-acid-etching. However, no dose-response in the corrosion behavior of the Ti-6Al-4V alloy could be observed. The capacitance of the double layer increased with the increased LPS concentration. These results suggest a greater susceptibility of Ti implant corrosion in diabetic patients with associated infections.
